# Changes Produced in the Nervous System by Civilization, Considered According to the Evidence of Physiology and the Philosophy of History

**Published:** 1838-04

**Authors:** 


### Art. XIV.

*Changes produced in the Nervous System by Civilization, considered according to the Evidence of Physiology and the Philosophy of History.* By Robert Verity, m.d. &c.—*London,* 1837. 8vo. pp. 79.

One of the first things that struck us, on taking up this production, was the disparity of dimension between the subject and the volume in which it is treated. The subject is one of great interest and of vast extent; it would, indeed, afford many years of occupation to the most powerful and comprehensive mind, aided by a wide range of historical, physical, and metaphysical learning; yet all that Dr. Verity has to say upon it is comprised within seventy-nine pages. In this small space, however, he contrived to pour out such a flood of words, that his reasoning, if such it can be called, is nearly drowned. If there were many such books, we should stand in need of a literary humane society for the recovery of submerged ideas.

The general scope of our author's argument is to demonstrate that the progress of civilization has been attended with a proportionate improvement in the organization of man, and especially in that of the nervous tissues; the cerebral functions acquiring increased power and activity as the moral and intellectual nature gains the ascendant over the animal.

sary to discuss. Dr. H. continues to take great credit to himself for having identified the new "motive power," which he regards himself as having been the first to demonstrate, with the *vis nervosa* of Haller; and for having rectified an important misstatement on the part of Haller and Professor Müller as to its propagation along the nerves. Now the fact appears to us to be, that Haller employed the term *vis nervosa* in precisely the sense which we and others have given to the term "motive influence," designating by it that organic change in the nervous fibre which is *originated* in the central organs of the nervous system, and which, by its *centrifugal* propagation, excites contraction of the muscular fibre. This motive influence may be produced either by an act of the will or more directly by a stimulus conveyed to the cerebro-spinal axis through the centripetal or afferent nerves; which stimulus, if further conveyed to the cerebrum, excites sensation. We regard Haller and Müller as perfectly correct, therefore, in stating that the *vis nervosa,* or *motor power,* acts only in the direction of the branches of the nerves. It is the stimulus by which this is excited in the central organs that is transmitted in a contrary direction. But, further, Dr. Hall is evidently wrong in the identification he has attempted; for, according to him, the excito-motor system is anatomically distinct from the cerebral or volitional, and therefore the motor power which acts through it cannot, according to his views, be the same as that derived from the brain, which organ is specially referred to by Haller as the originator of the *vis nervosa*. Dr. Hall might as well say that "motive power" is transmitted along the sensory nerves when an action is the result of the production of a *sensation* in the brain, as that it is transmitted along the afferent spinal nerves; for these only convey the impression by the stimulus of which to the spinal cord the change is produced in the efferent or motor nerves that constitutes motive power.

In order to make good this position, Dr. Verity takes a rapid survey of the partial and transient civilization of the great nations of antiquity; of the dark period that ensued, till the gradual commingling of races and of manners gave rise to more permanent principles of improvement; and of the influence of the Crusades, of chivalry, and other causes, which are generally acknowledged to have contributed to the formation of the present state of society. In his view of the character of these ages, and the operation of these causes, Dr. Verity does not appear to differ from the majority of philosophical historians, and for this very reason we think his observations entirely superfluous; the rather that his professed object is not to give a history of civilization, but to shew that civilization has produced a certain effect on the physical constitution of man. What is still more unfortunate, when he comes directly to the point of proof, he contents himself with telling us that the thing *must* have been so!

"As no human manifestation whatever can be allowed to take place without also its material conditions in the body, the physiologist knows well that the nervous system must have felt the influence of the general impulse and movement in the same full and equal degree as the historical evidence extends, and that the corresponding parts of the great nervous centres, from having been directly excited by this newly-created and additional activity for many generations, must have taken up, by successive accumulations of nutrition, an amplified form and size, permanently fixed in the organization, transmissible from parent to child, and forming, in short, an important step towards an improved character of type. The appearance of every new element of activity in the history of a race must ever be, to the physiologist, evidence of something added or modified in the nervous system. There is a rigid concatenation between the two circumstances." (P. 29.)

If physiologists know all this well, which we admit to be the case, to what purpose is the argument with which our author has been previously occupied? The truth is, he has been vainly seeking, in the history of past ages, for the evidences of a physiological fact which is amply demonstrated in the ascending scale of cerebral organization, as exhibited by modern anatomy, but on which history throws no light, because it is not the province of history to record the dissection of brains.

There are some other physical changes alleged by our author to result from civilization, concerning which we are very sceptical. Thus, we are told that "the bony structure, losing all excess of bulkiness and porosity, becomes denser, more compact, and more finely grained; gaining in strength and specific gravity what it loses in softness and clumsiness of size." (P. 59.) We should have liked a few examples of this sponginess of uncivilized bones; in the absence of which, we will give an example to the contrary that just now occurs to us. When the skeleton of Robert Bruce was discovered in 1819, it was examined by Professor Monro, who observes, "it was remarkable that even the very thin bones of the orbit were quite entire, as also all the processes of the bones at the base of the skull; even the pterygoid processes of the sphenoid bone and the palate bone."* The bones of the Scottish hero, then, must have been exceedingly well compacted, or their more fragile parts would not have lasted so long; yet he died in the comparatively rude era of 1350. We must further confess that the examinations we have made on various occasions of ancient bones, as compared with modern ones, and the bones of sa-

* Monro's Elements of Anatomy, vol. i. p. 21.

vages, as compared with those of civilized men, have not led us to the conclusion that their intimate texture is at all influenced by the degrees of civilization.

Dr. Verity, moreover, informs us that, "besides the amplified volume and enhanced temperament of the cerebral masses," which result from civilization, "the different structures of the body become interpenetrated with a more copious interlacement of nervous webbing, whereby all the complicated mechanism of animal and organic life is made to perform its various functions with more energy, more breadth, and more endurance." (P. 60.) This passage contains a most outrageous assumption. We admit that the sensibility of several of the textures is increased by circumstances connected with civilization; but we have yet to learn that this depends on an increased quantity of nervous matter interwoven with those textures; nor is there on record a single anatomical fact to bear out Dr. Verity's assertion.

Attendant upon all the progressive stages of civilization, great changes took place in diet, and the general hygienic circumstances, which, arising from increased moral refinement, reacted in their turn upon morals, through the medium of an improved physical organization. So says our author, though with greater copiousness of diction than we approve of or have room for. This, no doubt, is true to a certain extent: it is not to be forgotten, however, that gastronomy is the daughter of civilization.

Besides the matters above commented on, we find a proposal for a continued series of observations on the development of certain parts of the encephalon, in connexion with the social progress of man; a thing much to be desired, and to which phrenology, when placed on a more philosophical basis than it stands at present, will probably conduce. We find also certain precepts on the adaptation of temperaments and constitutions in marriage, which, we fear, can never be reduced to practice; and many observations about things in general, with which we do not think it needful to trouble the reader.

In the preface, Dr. Verity announces his intention of publishing, at some future period, "some views on the principles of medicine, considered in relation to the modified type of temperament produced by the increasing proportion of the nervous element in the organization of individuals who are fair representatives of the high civilization of modern times." To these views the present work is intended as an introduction; but, unless the author can bring himself to much severer habits of thinking, and a much denser style of writing, we would by all means dissuade him from the labour he contemplates.

Without at all knowing how the fact may be, we should strongly conjecture that Dr. Verity had read too many of those shallow and bombastic productions which encumber the fine field of German literature; for, although this book is written in the English language, it strikes us as essentially German in its style; and truly it yields not in feebleness and verbosity to the most inane productions of the German school.

